# Gene expression analysis of rheumatoid arthritis synovial lining regions by cDNA microarray combined with laser microdissection: up-regulation of inflammation-associated STAT1, IRF1, CXCL9, CXCL10, and CCL5

**DOI:** 10.3109/03009742.2011.623137

**Published:** 2012-03-09

**Authors:** S Yoshida, F Arakawa, F Higuchi, Y Ishibashi, M Goto, Y Sugita, Y Nomura, D Niino, K Shimizu, R Aoki, K Hashikawa, Y Kimura, K Yasuda, K Tashiro, S Kuhara, K Nagata, K Ohshima

**Affiliations:** 1 Department of Pathology, School of Medicine, Kurume University, Japan; 2 Department of Orthopaedic Surgery, School of Medicine, Kurume University, Japan; 3 Department of Orthopaedic Surgery, Kurume University Medical Centre, Japan; 4 Cell Innovator Inc., Venture Business Laboratory of Kyushu University, Japan; 5 Laboratory of Molecular Gene Technology, Department of Bioscience and Biotechnology, Faculty of Agriculture, Kyushu University, Japan

## Abstract

**Objectives:**

The main histological change in rheumatoid arthritis (RA) is the villous proliferation of synovial lining cells, an important source of cytokines and chemokines, which are associated with inflammation. The aim of this study was to evaluate gene expression in the microdissected synovial lining cells of RA patients, using those of osteoarthritis (OA) patients as the control.

**Methods:**

Samples were obtained during total joint replacement from 11 RA and five OA patients. Total RNA from the synovial lining cells was derived from selected specimens by laser microdissection (LMD) for subsequent cDNA microarray analysis. In addition, the expression of significant genes was confirmed immunohistochemically.

**Results:**

The 14 519 genes detected by cDNA microarray were used to compare gene expression levels in synovial lining cells from RA with those from OA patients. Cluster analysis indicated that RA cells, including low- and high-expression subgroups, and OA cells were stored in two main clusters. The molecular activity of RA was statistically consistent with its clinical and histological activity. Expression levels of signal transducer and activator of transcription 1 (STAT1), interferon regulatory factor 1 (IRF1), and the chemokines CXCL9, CXCL10, and CCL5 were statistically significantly higher in the synovium of RA than in that of OA. Immunohistochemically, the lining synovium of RA, but not that of OA, clearly expressed STAT1, IRF1, and chemokines, as was seen in microarray analysis combined with LMD.

**Conclusions:**

Our findings indicate an important role for lining synovial cells in the inflammatory and proliferative processes of RA. Further understanding of the local signalling in structural components is important in rheumatology.

Rheumatoid arthritis (RA) is a heterogeneous chronic autoimmune disease of the joints characterized by three main symptoms: inflammation, abnormal cellular and immune response, and synovial proliferation. Eventually, the interplay of these pathological processes leads to complete joint destruction (1). Factors that contribute to synovial hyperplasia are recruitment and retention of inflammatory cells, enhanced cell proliferation, and impaired apoptosis. Cytokines and chemokines, derived from activated synoviocytes, play an important role in the regulation of these processes (2, 3). Tumour necrosis factor (TNF)-α is a multifunctional cytokine that regulates the inflammatory reaction through the activation of various downstream genes. Synovial cells are one of the targets of TNF-α, which is recognized as a key molecule in the pathogenesis of RA (4). Several studies (5–7) have reported that signal transducer and activator of transcription 1 (STAT1) expression is abundant in RA synovial tissue compared with controls, indicating that the STAT1 pathway is activated by phosphorylation. Yarilina et al (8) described the delayed and chronic inflammatory responses induced by the TNF-activated interferon regulatory factor 1 (IRFl)-interferon (IFN)-STAT1 pathway. In addition, STAT1 is thought to be essential for IFN signalling (8, 9). IFNs are typically growth inhibitory and promote the immune recognition of target cells as well as activating STAT signalling, which sustains inflammatory chemokine expression. The absence of IFN/STAT1 signalling has been associated with an increase in susceptibility to infection and tumour formation (10).

Chemokines are a family of small proteins that regulate cell migration into sites of inflammation (11). An important advance in understanding the development and progression of RA was the recognition that chemokines expressed in the synovium of RA joints are key mediators in the pathogenesis of RA synovitis (12–16).

Morphologically, rheumatoid synovium contains several cellular components, including fibroblasts, macrophages, and lymphocytes, and is composed structurally of a lining layer, a sublining, vessels, and lymphoid follicles with cells that show a variety of gene expressions.

Complementary DNA (cDNA) microarray technology constitutes a powerful way to gain insight into the molecular complexity and pathogenesis of arthritides (17–24), and makes it possible to identify the differences in numerous gene expressions (5, 25). However, microarray findings in RA have been unclear because rheumatoid synovial tissue is characterized by infiltration of the sub-lining by a combination of macrophages, plasma cells, T and B cells, and other inflammatory cells that promote inflammation. Gene expression in each tissue compartment should therefore be analysed separately to gain a better understanding of the contribution of each component of a tissue to the pathogenesis of RA. To overcome this problem, laser microdissection (LMD) was used in this study. LMD combined with extraction of total RNA followed by cDNA microarray is a technique that has been developed mainly for molecular oncology and is used for identifying molecular markers of selected tumour cells (26, 27). The use of this combination technique for the analysis of gene expression in synovial lining cells in RA and disease progression should improve our understanding of RA at the molecular level.

## Materials and methods

### Patients and tissue samples

Human synovial samples were obtained during total joint replacement surgery from 11 patients who met the American College of Rheumatism revised criteria for RA (28). The control synovial samples were obtained from the knee and hip joints of five radiologically diagnosed cases of osteoarthritis (OA) during total joint replacement. All patients were hospitalized and treated at Kurume University Hospital and Kurume University Medical Centre, Japan. The clinical, biological, and demographic data for all patients as well as their laboratory data obtained from the medical records at the two institutions are summarized in Table 1. Clinical activity was classified into high (H) or low (L) on the basis of findings for rheumatoid factor (RF), C-reactive protein (CRP), and the symptom criteria for RA. Krenn's syno-vitis score was used for histological determination of RA activity. This scoring system (range: 0–9) is a simple histopathological classification for grading three features of inflammation in synovial tissue to establish the syno-vitis score: 0–1, no synovitis; 2–4, low-grade synovitis, and 5–9, high-grade synovitis (29). In Table 1, 4–6 corresponds to L (low RA activity) and 7–8 to H (high RA activity). This study was approved by the Kurume University Institutional Review Board, and patients provided informed consent in accordance with the Declaration of Helsinki. Written consent for participation in this study was obtained from all the patients.

**Table 1:** Clinical and laboratory data of the patients with RA and OA who were studied for gene expression profiling in synovial tissue.

				Clinical activity			Histological activity		
						
Case	Diagnosis	Age (years)	Sex	RF	CRP (mg/L)		Krenn		Molecular activity
S17	RA	63	F	+	14	H	8	H	H
S20	RA	64	M	+	36	H	8	H	H
S49	RA	54	F	+	30	H	7	H	H
S52	RA	85	F	ND	18	H	7	H	H
S56	RA	62	F	+	42	H	6	H/L	H
S57	RA	79	F	+	24	H	7	H	H
S68	RA	73	F	+	127	H	7	H	H
S69	RA	52	F	+	22	H	9	H	H
S11	RA	73	M	+	0	L	5	L	L
S53	RA	78	F	ND	0	L	4	L	L
S65	RA	71	F	ND	0	L	5	L	L
S13	OA	68	F	–	0		3		
S40	OA	79	F	–	0		2		
S62	OA	76	M	–	0		2		
S63	OA	63	F	–	0		2		
S66	OA	75	F	–	2		3		

RF, Rheumatoid factor; CRP, C-reactive protein; Krenn, synovitis score; H, high inflammation RA; L, low inflammation RA; ND, not determined.

### LMD

**Figure 1:**
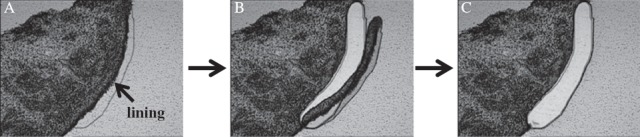
Microdissection of the synovial lining in RA. (A) After toluidine blue staining, a section was subjected to LMD. The sample was observed on the PC monitor and the lining area was marked. (B) The sample was cut with a laser beam along the defined line. (C) After microdissection, the sample was collected by gravity into a tube cap. In this manner, we obtained more than 5000 cells from the RA or OA synovial lining sections.

The tissue samples were immediately frozen in acetone/dry ice and stored at −80°C for microdissection. The synovial samples were embedded in an optical cutting temperature (OCT) compound (Sakura Finetek, Tokyo, Japan) and frozen in liquid nitrogen. Cryosections (20-μm-thick) were mounted on 2.0-μm-thick PEN-Membrane slides (MicroDissect GmbH, Herborn, Germany). After fixation in 100% ethanol, the slides were stained rapidly with toluidine blue O (Chroma-Gesellschaft Schmid GmbH & Co, Köngen, Germany) and then washed in diethylpyro-carbonate (DEPC)-treated water and air-dried with a fan. The frozen sections were microdissected with a Leica LMD6000 laser microdissection system following the company's protocol (Leica, Wetzlar, Germany). The synovial lining regions were microdissected from the tissue sections with LMD (Figure 1), and the dissected cells were collected in 0.5 mL tube caps filled with 50 μL lysis buffer for RNA extraction (29, 30).

### RNA extraction and biotinylated cRNA amplification

Total RNA was extracted from the samples collected by means of LMD and with an RNAqueous-Micro kit (Ambion, Austin, TX, USA) according to the manufacturer's instructions. cRNA amplification and labelling with biotin were used for gene expression profiling by microarray analysis. In brief, 500 ng total RNA was amplified overnight (14 h) with the Illumina Total Prep RNA Amplification kit (Ambion) in accordance with the manufacturer's protocol. Reaction cRNA was biotinylated during in vitro transcription.

### Illumina BeadChips microarray

Sentrix Human WG-6 v3.0 Expression BeadChips were purchased from Illumina, Inc (San Diego, CA, USA). More than 48 000 different bead types, each with a 50-base gene-specific probe, are represented on a single BeadChip. For each probe represented on the array, beads are assembled with an average 30-fold redundancy. A hybridization mixture containing 1.5 μg biotinylated cRNA was hybridized to the BeadChips at 58°C overnight (18 h) before being washed and stained with streptavidin-Cy3 (GE Healthcare, Buckinghamshire, UK) according to the manufacturer's instructions. BeadChips were scanned on Illumina BeadStation 500 and fluorescent hybridization signals were assessed with Illumina BeadStudio software.

### Statistical analysis

For the preprocessing step, variances in the data were first stabilized with the variance stabilizing transform (VST) method (32) and then normalized with a robust spline normalization method, both of which are used in the Lumi BioConductor package (Illumina) (33). To reduce false positives, effectively absent transcripts were filtered out. Transcripts were considered detected if the detection p-value calculated from the background with the Illumina BeadStudio was less than 0.05 for all hybridizations. The Significance Analysis of Microarrays (SAM) statistical test (34), which is used in the BioConductor ‘samr’ package and takes multiple testing into account by estimating the false discovery rate (FDR), was used to identify differentially expressed transcripts in RA patients. If a transcript was up- or down-regulated by a factor of 2 or more and had a SAM q-value (FDR) of less than 0.001, we regarded this transcript as differentially expressed in RA patients.

To obtain reproducible clusters for classifying the 16 samples from the 11 RA and 5 OA patients, expression data were analysed with GeneSpring 7.2 software (Silicon Genetics, Redwood City, CA, USA), which was also used to generate heatmaps of certain genes of interest. Ingenuity Pathway Analysis (IPA6.0; Ingenuity Systems, Redwood, CA, USA; http://www.ingenuity.com) was used to identify networks of interacting genes. Lists of expressed (up- and down-regulated) genes were uploaded for IPA.

### Immunohistochemistry and microscopic analysis

The synovium specimens (25 RA and 10 OA) were fixed in buffered formalin, embedded in paraffin, and then stained with haematoxylin–eosin. The immunohisto-chemical staining of STAT1 (BD Biosciences, Franklin Lakes, NJ, USA), CCL5, CXCL9, CXCL10 (R&D Systems, Inc, Minneapolis, MN, USA), and IRF1 (Abeam Inc, Cambridge, MA, USA) was performed in a single laboratory, as described previously (35). For immunohistochemical analysis, the expression of STAT1 and CCL5 was scored on a four-point scale as follows: grade 0: 0–10% of the synovial lining cells stained; grade 1: 10–40% stained; grade 2: 40–70% stained; grade 3: > 70% stained. Clinical and pathological findings for different groups were compared using the Student t test and the χ^2^ test. Values of p < 0.05 were considered significant.

## Results

### Gene-expression profiles and clustering

**Figure 2:**
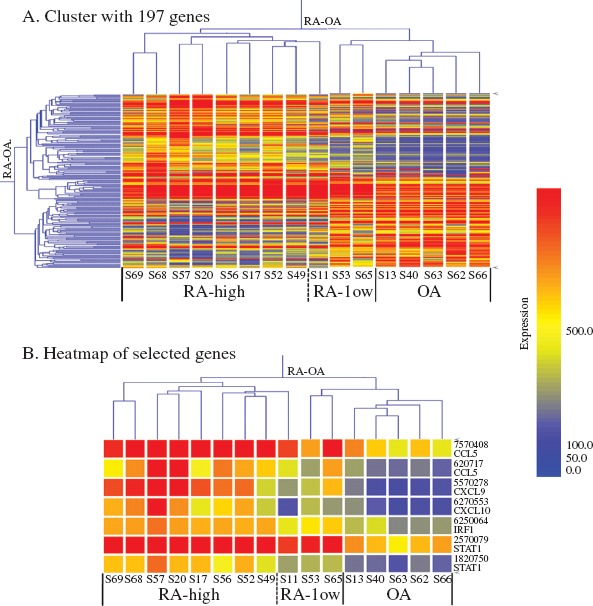
Gene-expression clustering in RA and OA. (A) Cluster with 197 genes (after SAM analysis of normalized 14 519 genes; criterion: FC RA/OA ≥ 2.0 or ≤ 0.5, FDR < 0.001). The dendrogram shows the major groups, that is RA (subgroups: high RA and low RA) and OA groups. (B) Heatmap of selected genes. Expression of chemokines (CCL5 and CXCL9/10) and transcription factors (IRF1 and STAT1) were selected and analysed using Gene-Spring software. The levels of these genes are more strongly up-regulated in RA than in OA. The colour intensity indicates the magnitude of the deviation from the median (yellow). Red represents relative expression levels higher than the median level across all tissues and blue represents lower expression levels.

With the Illumina BeadStudio software, we detected 14 519 genes that showed significant differences between the RA and OA groups, and with SAM statistical analysis 197 genes were selected, both up-regulated (n = 121) and down-regulated (n = 76). Based on their gene-expression profiles (Figure 2A), the 16 synovium samples were divided into two major groups, RA and OA. The RA samples were clinically further divided into two subgroups (RA-high and RA-low) based on their hierarchical clustering patterns and the clinical data of CRP values and histo-logical Krenn scores as shown in Table 1. The result of this clustering profile indicated that the gene expression patterns in RA tissues were heterogeneous. Of the 16 cases, eight (S17, 20, 49, 52, 56, 57, 68, and 69) were clustered into the RA-high and three (S11, 53, and 65) into the RA-low subgroup, and the remaining five cases (S13,40,62,63, and 66) were clustered into the OA group (Figure 2A). Molecular activity presented in Table 1 shows the clustering results, with H and L indicating RA-high and RA-low, respectively. Each group appeared to have a specific gene profile that could be related to the molecular nature of their aetiological differences.

### Genes up- and down-regulated in RA

**Figure 3:**
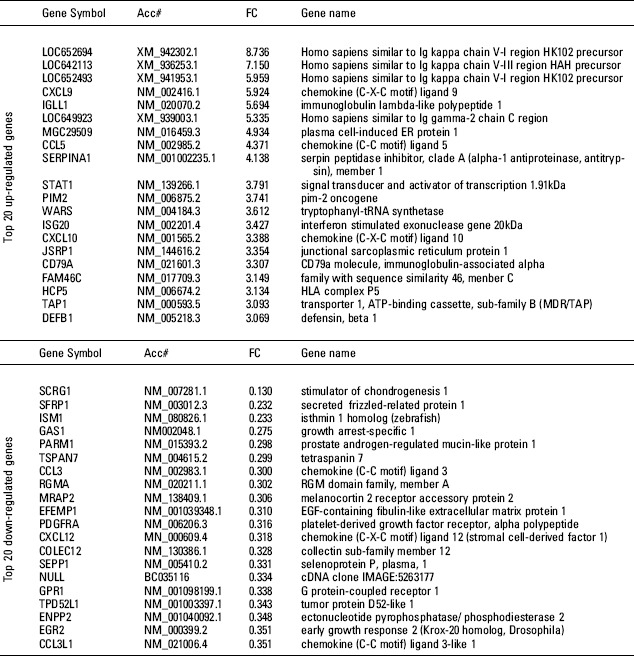
List of top 20 up- and down-regulated genes from the filtered 197 genes. The upper panel shows the top 20 genes from 121 up-regulated genes (RA/OA) on the basis of Fold Change (FC). The lower panel is a list of the bottom 20 genes from 76 down-regulated genes.

IPA analysis of the filtered 197 genes revealed that 48 genes belonged to the category of inflammatory response function. The 48 genes were: AIM2, ALOX5, ALPP, ANPEP, APP, AQP9, CCL3, CCL3L1, CCL5, CD2, CD247, CD79A, CD8A, CFB, CFH, COLEC12, CTGF, CXCL9, CXCL10, CXCL12, DEFB1, EGR2, FGR, GZMA, GZMH, HCP5, HLA-B, HLA-DQB1, ICAM3, IRF1, ISG20, ITGAX, LILRB2, LTB, PDE4B, PROS1, RAC2, S100A8, SEMA4D, SERPINA1, SLAMF6, STAT1, TAP1, TAPBP, and TGFBR3. Of those genes, CCL5, CXCL9, CXCL10, STAT1, CD79A (CD79a molecule, immunoglobulin-associated alpha), DEFB1 (defensin, beta 1), HCP5 (HLA complex P5), ISG20 (IFN-stimulated exonuclease gene 20 kDa), and TAP1 (transporter 1, ATP-binding cassette, subfamily B) were included in the top 20 up-regulated genes on the basis of Fold Change (FC). IRF1, which functioned as transcriptional activator of IFNβ, was also up-regulated in RA. Conversely, the genes listed in the bottom 20 were CCL3, CCL31, and CXCL12 (Figure 3). In this analysis, we focused on chemokines and transcription factors, especially STAT1 and IRF1.

**Figure 4:**
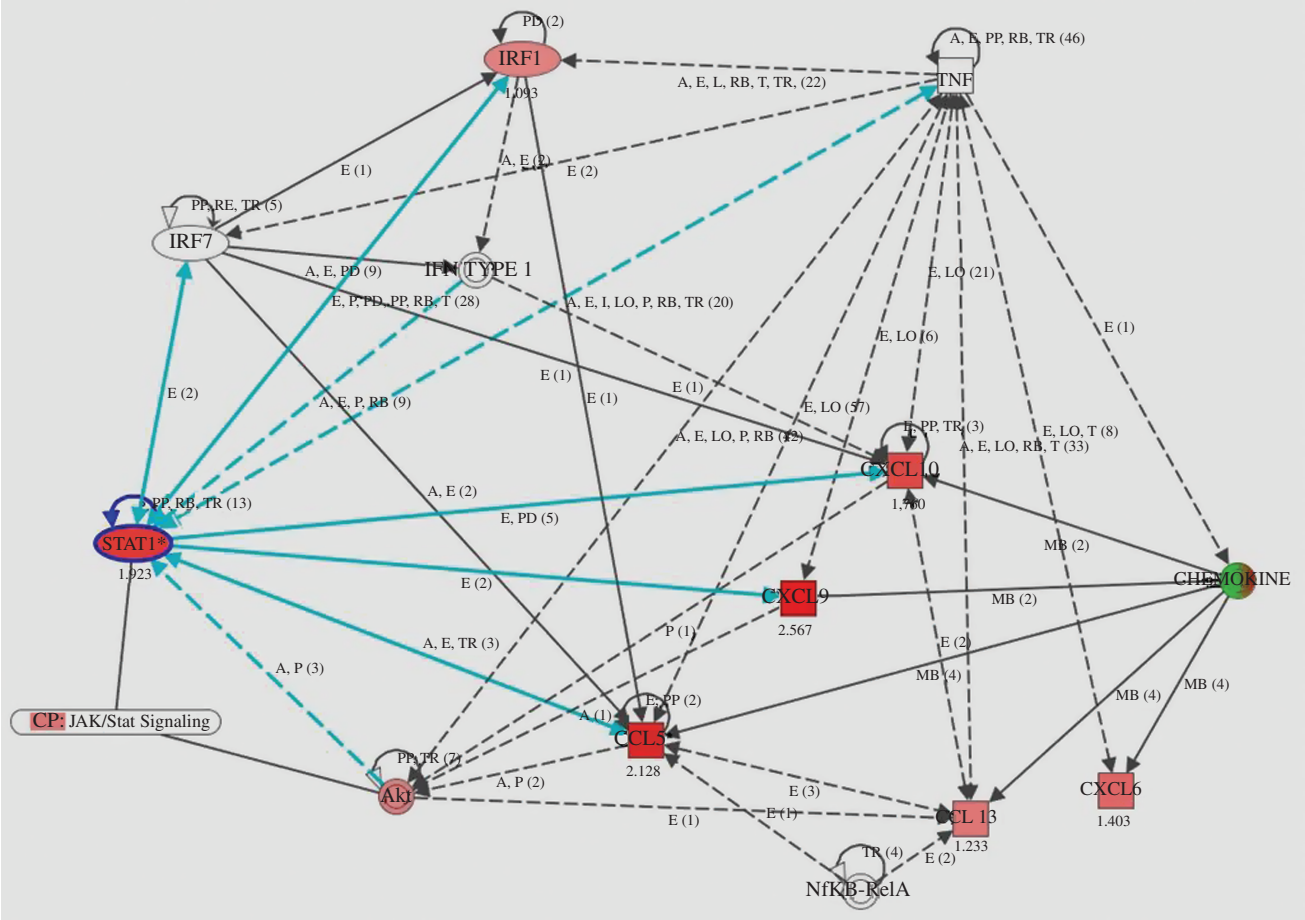
Gene network generated through the use of Ingenuity Pathway Analysis (IPA). IPA was applied to TNF, chemokines (CCL5, CXCL9/10), and transcription factor (STATl and IRFl) genes to create a network inter-related to inflammatory mediator genes. Interacting nodes are defined by either direct relationships, which require direct physical contact (solid arrows), or indirect relationships (dashed arrows). The direction of the arrows shows the direction of the interaction. The blue line indicates the connection with STATl. Red molecules indicate higher expression in RA than in OA at the gene level.

As seen in Table 2, CCL5, CXCL9, and CXCL10 were up-regulated more in the RA than in the OA group (FC > 2.0), while CXCL9, CCL5, and CXCL10 levels showed a more than 3-fold increase in the RA samples. On the contrary, CCL3, CCL3L1, and CXCL12 were down-regulated less in the RA group (FC < 0.5). In addition, gene expression in RA showed the following rates of up-regulation: STAT1β 3.79-fold; STAT1α 2.09-fold; and IRF1 2.13-fold. Up-regulation of STAT1β, which is the splice variant of full-length STAT1α, was higher than that of STAT1α, but the former lacked a C-terminus containing Ser727. At the protein level, however, the phosphorylation of Ser727 is not required for activation of the signal-transduction pathways by type I IFN (36, 37). Moreover, the heatmap in Figure 2B shows that, in comparison with OA, levels of CCL5, CXCL9/10, STAT1, and IRF1 in RA were strongly up-regulated. On the basis of these data, the pathway analysis of inflammatory responses of RA was performed. The network created by IPA shows that TNF, IRF1, and type I IFN are connected to CCL5, and to CXCL9/10 through STAT1 (Figure 4).

**Table 2:** List of up- and down-regulated genes (chemokines and transcription factors).

				RA vs. OA	
				
Probe ID	Gene symbol	Accession no.	Gene name	Fold Change	p-value
Chemokine					
5570278	*CXCL9*	NM_002416.1	Chemokine (C-X-C motif) ligand 9	5.92	0.0001
7570408	*CCL5*	NM_002985.2	Chemokine (C-C motif) ligand 5	4.37	0.0002
620717	*CCL5*	NM_002985.2	Chemokine (C-C motif) ligand 5	3.39	0.0005
6270553	*CXCL10*	NM_001565.2	Chemokine (C-X-C motif) ligand 10	3.39	0.0016
4250053	*CCL3L1*	NM_021006.4	Chemokine (C-C motif) ligand 3-like 1	0.35	0.0014
4610615	*CXCL12*	NM_000609.4	Chemokine (C-X-C motif) ligand 12 (stromal cell-derived factor 1)	0.32	0.0004
6590682	*CCL3*	NM_002983.1	Chemokine (C-C motif) ligand 3	0.30	0.0013
Transcription factor					
2570079	*STAT1*	NM_139266.1	Signal transducer and activator of transcription 1.91 kDa (β-type)	3.79	0.0001
1820750	*STAT1*	NM_007315.2	Signal transducer and activator of transcription 1.91 kDa (common)	2.56	0.0001
4810187	*STAT1*	NM_007315.2	Signal transducer and activator of transcription 1.91 kDa (α-type)	2.09	0.0001
6250064	*IRF1*	NM_002198.1	Interferon regulatory factor 1	2.13	0.0001

### Immunohistochemistry

**Figure 5:**
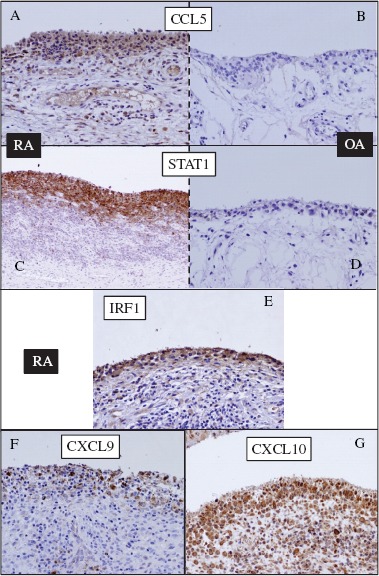
Immunohistochemical staining of the synovium from (A, C, E, F, and G) RA and (B and D) OA patients. (A and B) anti-CCL5; (C and D) anti-STATl; (E) anti-IRFl; (F) anti-CXCL9; (G) anti-CXCLlO. Significant presence of all antigens was detected in the synovial lining cells of RA but not of OA.

In an independent study using synovium from patients with OA and RA, we could confirm the existence of differential expressions of STAT1, CCL5, CXCL9/10, and IRF1 at the protein level (Figure 5). Immunohistochemical analysis revealed significantly high expressions of STAT1, CCL5, CXCL9/10, and IRF1 in the synovial lining cells of RA, but not of OA. CCL5, CXCL9/10, and IRF1 were generated in the cytoplasm of the synovial lining cells. Although IRF1 is a transcription factor, its abundant expression was clearly observed in the cytoplasm of lining cells in RA. STATl was found to be generated in the nucleus and cytoplasm. Of note, strong inflammatory activity associated with infiltration of lymphocytes showed strongly positive staining for STATl and CCL5 (data not shown).

**Figure 6:**
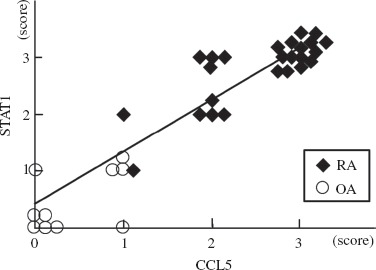
Relationship between STAT1 and CCL5 established by immunohistochemical analysis. Expression of STAT1 and CCL5 at the protein level is higher in the synovial cells of RA (n = 25) than in those of OA (n = 10). The STAT1 score correlates significantly with the CCL5 score (r = 0.93, p < 10^−6^).

Because of strong staining in the RA synovial lining regions, we investigated whether there was a relationship between STATl and CCL5 (Figure 6). We found that expression of STATl and CCL5 in the synovial cells of RA patients (n = 25) was higher than in OA patients (n = 10), while a statistically significant correlation between STAT1 and CCL5 was confirmed (r = 0.93, p < 10^−6^). Consistent with the molecular profiling data, positive cells were predominantly found in the synovial lining layer of RA.

### Relationship between molecular profiles and disease parameters

We next investigated whether the molecular definitions of the RA tissue subgroups were associated with clinical differences between RA patients. First, the relationship between molecular definitions and parameters of clinical and demographic data was determined (Table 1). For CRP and Krenn classification, a statistically significant difference was observed between patients whose tissues featured histologically identified fulminating inflammation in the form of lymphofollicles and synovial palisading cells (high-RA group: H) and those whose tissues featured mild inflammation (low-RA group: L) (p < 0.008; t-test). The mean Krenn score for group H (7.38) was significantly higher than that for group L (4.67), as was the mean of CRP (39.125 and 0, respectively). The use of microarray combined with LMD of the synovial lining layer in this study thus made it more evident that the clinical parameters that are subject to disease activity of RA in systemic mechanisms are closely associated with molecular profiles.

## Discussion

Disease progression of RA, which is characterized by villous proliferation of synoviocytes, mainly of synovial fibroblasts, results in bone and joint destruction. The spontaneous arrest of the proliferation of synovial tissue is of particular interest, strongly suggesting the involvement of apoptosis in this process. Villous proliferation involves both the proliferation and apoptosis of synovial lining cells, the latter being affected by various T-cell types, primarily cytotoxic T-lymphocytes (CTLs), which infiltrate the synovial lining cell layer. The activated T cells attack and damage the synovial lining cells, causing apoptosis and leading to the growth of new synovial cells to replace the damaged cells (38–40). The synovial lining layer samples used for the microarray analysis were dissected synovial tissues without joint erosion and bone tissue.

We used LMD followed by a cDNA microarray to analyse gene-expression profiles in the synovial lining tissues in RA. A total of 14519 genes from among more than 48000 transcripts met the normalization criteria for microarray analysis and were divided into an RA group and an OA group. After filtering with the SAM method, detailed expression profiles of the 197 genes statistically selected from among all the cases were obtained. Cluster analysis produced two distinct molecular RA subgroups (high RA and low RA), which were closely associated with the histological and clinical activity of RA. The two molecular subgroups showed different expression levels of genes crucial for proliferative inflammation. The expressions of CCL5, CXCL9/10, STAT1, and IRF1 were found to be up-regulated in RA. Because of the small quantity of total RNA obtained from the dissected sample, quantitative real-time transcription polymerase chain reaction (qRT-PCR) assays were performed using the same cRNA samples as for the microarray analysis. The qRT-PCR measurements were always very similar to the expression levels of the microarray (data not shown). In our study, the results of immunohistochemical analysis of the up-regulated molecules, that is CCL5, CXCL9/10, STAT1, and IRF1, matched the results of the analysis of mRNA expression levels.

The various cytokines and chemokines present in the RA synovium create a complex situation with simultaneous activation of multiple signalling pathways that may influence STAT1 signalling. As a result, the synovium becomes inflamed. Yarilina et al (8) reported that TNF initiated a type I IFNβ-mediated autocrine loop and that expression of inflammation-related genes (CXCL9, CXCL10, CCL5, etc.) was sustained and amplified by the sequential induction of IRF1, IFNβ, and STAT1. TNF-mediated production of IFNβ and the ensuing autocrine regulation of gene expression was shown to depend on IRF1 and on synergy between the small amounts of IFNβ produced and additional TNF-induced signals, such as activation of NF-*k*B. The model used by Yarilina et al, the TNF-activated IRF1-IFNβ-JAK-STAT signalling pathway, contributes to the proinflammatory functions of TNF and the macrophage responses to endogenous inflammatory factors such as TNF (8). In our study, strong expression of CCL5, CXCL9/10, STAT1, and IRF1 was observed in the RA synovial lining cells at mRNA and protein levels. IFNβ could not be detected by the normalization criteria, but it is possible that its expression at the mRNA level is slight, which make it possible to maintain the autocrine loop. Several studies have reported that the IFNβ protein was observed in synovial tissue of RA rather than of OA and was detected in all compartments of the synovium, especially in fibroblast-like synoviocytes (FLS) of the intimal lining layer (41, 42). In our study, the expression of IFNβ in RA synovial tissue was detected by means of immunohistochemical staining (data not shown). As shown in Figure 4, the network created by IPA suggested that the TNF-activated IRF1-IFN-STAT1 pathway is located in the synovial lining cells of RA. This finding in our study supports the notion that the expressions of molecules contained in the model, that is, in the TNF-mediated autocrine and feedforward loop, are highly similar. In addition, the local signalling seemed to be impacted by synovial proliferation.

Nowadays, RA is often treated with TNF inhibitor therapy, but this treatment is associated with side-effects on the systemic immune system and may cause serious complications such as tuberculosis and malignancy (43, 44). However, we found in this study that IRF1 appears to be clinically efficacious for the inhibition of chronic and delayed inflammation of RA, and that target therapy using IRF1 may entail fewer immunogenic systemic immunodeficiency problems than do therapies using TNF antibodies. Moreover, our results indicate that treatment using the IRF1 antibody would help to inhibit the autocrine loop, which is associated with the TNF-activated IRF1-IFNβ-JAK-STAT signalling pathway. One study has in fact suggested that inhibition of the IRF1 transcription factor may represent a novel approach to controlling RA (45).

Chemokines and their receptors play important roles in directing the migration of immunocompetent cells to sites of inflammation and determining the pathohistological outcome of chronic inflammation and synovial hyper-plasia (46, 47). Serum concentrations of CXCL9/10 may thus serve as sensitive markers for disease activity in patients with RA (48). It has been reported that CXCR3, the receptor of CXCL9/10, was strongly expressed by mast cells within the sublining region of RA synovial tissues. These findings suggest that the presence of CXCR3 protein on mast cells in RA sublining synovial tissue plays a significant role in the pathophysiology of RA, and is accompanied by elevated levels of CXCL9/10 (49). In other studies, CXCL9/10 have been shown to be highly expressed in RA synovial tissues and fluids (49–52), and the concentration gradient of CXCL9/ 10, between the serum and synovial fluid, favours the migration of CXCR3 receptor-expressing cells from the blood into synovium in RA (50). Our data show that expression of CXCL9/10 at the protein level was higher in the RA synovial lining region than in the sublining. The synovial lining cells thus play an important role in the production of CXCL9/10.

CXCL12 in RA has been shown to play multifunctional roles in the recruitment, retention, and survival of inflammatory cells as well as in angiogenesis and joint tissue destruction. In human synovial fibroblast cell lines, the CXCL12 gene is up-regulated at the transcriptional level by growth arrest, suggesting that it is associated with synovial proliferation (53). Using cDNA microarray from bulk tissues, van der Pouw Kraan et al (5) showed that the expression of the CXCL12 gene was up-regulated in RA synovium in comparison with OA tissues. By contrast, we found that the expression of the CXCL12 gene was down-regulated in the RA synovial lining region, suggesting that it was transcribed by sublining cells of RA at a higher level. In fact, an increase in CXCL12 immunos-taining has been observed in the RA sublining synovium and in pervascular inflammatory aggregates as compared with OA (54).

Our study indicates that the findings from the micro-array analysis of RA synovial lining cells are similar to the gene expression patterns obtained from RA bulk tissues (5); that is, STAT1, IRF1, CCL5, and CXCL9/10 were highly expressed in the synovial lining cells of RA. We also demonstrated the presence of molecularly distinct classes of the rheumatoid synovium.

In conclusion, we have analysed gene-expression profiles in the synovial lining tissues in RA and OA, followed by LMD and cDNA microarray analyses, and found that the molecular activities correspond to their clinical and histological counterparts. As for synovial proliferation, our findings point to the importance of a mechanism in which TNF-induced gene expression is sustained and amplified as a result of the sequential induction of IRF1, IFNβ, STAT1, and the chemokine pathway in the synovial lining layer of RA. Finally, the different expression profiles of several candidate genes identified in our study may provide useful information for future studies using the LMD technique concerning the diagnosis and prognosis of RA. Our study has made it clear that it is important in rheumatology to understand the local signalling of every structural component, such as the lining, sublining layer, and lymphoid follicles.
